# Supplementary material for: Abnormality prediction and forecasting of laboratory values from electrocardiogram signals using multimodal deep learning
Source: Sci Rep. 2025 Nov 10;15:39362. doi: 10.1038/s41598-025-26715-5 (PMC12603147; doi:10.1038/s41598-025-26715-5)
Supplement: Supplementary file 1 — Supplementary Information. [file 41598_2025_26715_MOESM1_ESM.pdf]

Predictive performance at operating point

Table S1. Sensitivity (sens.) and specificity (spec.) at sensitivity theshold 0.75 for the labels investigated in the main manuscript across the task laboratory values abnormality prediction. Next to each value name, we highlight the physiological category it represents: Cardiac (Ca.), Renal (Re.), Hematological (He.), Metabolic (Me.), Immunological (Im.), and Coagulation (Co.). The values in bold represent the highest performance per abnormality. For PT at the 30 minute horizon no score was estimated as it did not satisfy the inclusion criterion of at least 10 abnormal cases in both test and validation set.

Value	Threshold	Sensitivity/Specificity
NTproBNP [Ca.]	≥353 pg/mL	0.751/0.881
Hemoglobin [He.]	≥17.5 g/dL	0.750/0.822
Albumin [Me.]	≥5.2 g/dL	0.750/0.742
Acetaminophen [Me.]	≥30 ug/mL	0.768/0.839
Hematocrit [He.]	≥51%	0.750/0.761
Red Blood Cells [He.]	≥6.1 m/uL	0.750/0.699
Urea Nitrogen [Re.]	≤6 mg/dL	0.750/0.696
Creatinine [Re./Ca.]	≥1.2 mg/dL	0.750/0.675
Bilirubin, Direct [Me.]	≥0.3 mg/dL	0.752/0.638
Urea Nitrogen [Re.]	≥20 mg/dL	0.750/0.674
Albumin [Me.]	≤3.5 g/dL	0.750/0.642
Hemoglobin [He.]	≤13.7 g/dL	0.750/0.651
Cholesterol HDL [Me.]	≤41 mg/dL	0.755/0.676
RDW-SD [He.]	≥46.3 fL	0.751/0.654
Absolute Basophil Count [Im.]	≤0.01 K/uL	0.750/0.618
Creatinine [Re./Ca.]	≤0.5 mg/dL	0.750/0.657
Red Blood Cells [He.]	≤4.6 m/uL	0.750/0.606
RDW [He.]	≥15.5%	0.750/0.609
Hematocrit [He.]	≤40%	0.750/0.651
INR PT [Co.]	≥1.1 ratio	0.750/0.583
Bilirubin, Total [Me.]	≥1.5 mg/dL	0.750/0.581
PT [Co.]	≥12.5 sec	0.750/0.564
Lymphocytes [Im.]	≤18%	0.750/0.564
MCHC [He.]	≤31 g/dL	0.750/0.562

Table S2. Sensitivity (Sens.) and specificity (Spec.) at sensitivity theshold 0.8 for the labels investigated in the main manuscript across the task laboratory values abnormality prediction. Next to each value name, we highlight the physiological category it represents: Cardiac (Ca.), Renal (Re.), Hematological (He.), Metabolic (Me.), Immunological (Im.), and Coagulation (Co.). The values in bold represent the highest performance per abnormality. For PT at the 30 minute horizon no score was estimated as it did not satisfy the inclusion criterion of at least 10 abnormal cases in both test and validation set.

Value	Threshold	Sens./Spec.	Sens./Spec.	Sens./Spec.
NTproBNP [Ca.]	≥353 pg/mL	0.753/0.860	0.751/0.884	0.751/0.907
Hemoglobin [He.]	≥17.5 g/dL	0.750/0.828	0.750/0.886	0.750/0.815
Hematocrit [He.]	≥51%	0.750/0.768	0.750/0.744	0.755/0.719
Urea Nitrogen [Re.]	≤6 mg/dL	0.750/0.696	0.750/0.747	0.750/0.789
Creatinine [Re./Ca.]	≥1.2 mg/dL	0.750/0.692	0.750/0.705	0.750/0.709
Urea Nitrogen [Re.]	≥20 mg/dL	0.750/0.698	0.750/0.701	0.750/0.701
Red Blood Cells [He.]	≥6.1 m/uL	0.750/0.669	0.750/0.636	0.750/0.572
Hemoglobin [He.]	≤13.7 g/dL	0.750/0.667	0.750/0.662	0.750/0.666
Albumin [Me.]	≤3.5 g/dL	0.750/0.650	0.750/0.664	0.750/0.664
RDW-SD [He.]	≥46.3 fL	0.751/0.652	0.751/0.674	0.750/0.648
Absolute Monocyte Count [Im.]	≤0.2 K/uL	0.750/0.623	0.750/0.667	0.750/0.626
RDW [He.]	≥15.5%	0.750/0.624	0.750/0.631	0.750/0.631
Absolute Lymphocyte Count [Im.]	≥3.7 K/uL	0.750/0.601	0.750/0.653	0.750/0.548
Red Blood Cells [He.]	≤4.6 m/uL	0.750/0.636	0.750/0.619	0.750/0.612
C-Reactive Protein [Im.]	≥5 mg/L	0.756/0.520	0.750/0.618	0.750/0.673
Hematocrit [He.]	≤40%	0.750/0.635	0.750/0.622	0.750/0.612
Absolute Basophil Count [Im.]	≤0.01 K/uL	0.75/0.547	0.75/0.649	0.75/0.630
INR PT [Co.]	≥1.1 ratio	0.751/0.576	0.750/0.587	0.750/0.585
Lymphocytes [Im.]	≥42%	0.750/0.617	0.750/0.600	0.750/0.603
PT [Co.]	≥12.5 sec	0.750/0.567	0.750/0.570	0.750/0.567
Lymphocytes [Im.]	≤18%	0.750/0.600	0.750/0.578	0.750/0.588
Anion Gap [Me.]	≥20 mEq/L	0.750/0.611	0.750/0.578	0.750/0.568
MCHC [He.]	≤31 g/dL	0.750/0.595	0.750/0.590	0.750/0.577
Bilirubin Total [Me.]	≥1.5 mg/dL	0.750/0.540	0.750/0.569	0.750/0.590

Label prevalence and sample count

Table S3. Label prevalence and sample count for the labels investigated in the main manuscript across the tasks laboratory values abnormality prediction. Next to each value name, we highlight the physiological category it represents: Cardiac (Ca.), Renal (Re.), Hematological (He.), Metabolic (Me.), Immunological (Im.), and Coagulation (Co.). The values in bold represent the highest performance per abnormality. For PT at the 30 minute horizon no score was estimated as it did not satisfy the inclusion criterion of at least 10 abnormal cases in both test and validation set.

**Value**	**Threshold**	**Label prevalence**	**Sample count**
NTproBNP [Ca.]	≥353 pg/mL	77.72%	23,206
Hemoglobin [He.]	≥17.5 g/dL	72.39%	236,802
Albumin [Me.]	≥5.2 g/dL	0.4%	57,040
Acetaminophen [Me.]	≥30 ug/mL	18.76%	1,205
Hematocrit [He.]	≥51%	0.58%	238,567
Red Blood Cells [He.]	≥6.1 m/uL	0.31%	234,079
Urea Nitrogen [Re.]	≤6 mg/dL	0.89%	240,892
Creatinine [Re./Ca.]	≥1.2 mg/dL	28.52%	241,968
Bilirubin, Direct [Me.]	≥0.3 mg/dL	57.24%	3,438
Urea Nitrogen [Re.]	≥20 mg/dL	41.6%	240,892
Albumin [Me.]	≤3.5 g/dL	22.5%	57,040
Hemoglobin [He.]	≤13.7 g/dL	72.39%	236,802
Cholesterol HDL [Me.]	≤41 mg/dL	23.31%	9,023
RDW-SD [He.]	≥46.3 fL	49.16%	73,001
Absolute Basophil Count [Im.]	≤0.01 K/uL	7.81%	51,315
Creatinine [Re./Ca.]	≤0.5 mg/dL	1.03%	241,968
Red Blood Cells [He.]	≤4.6 m/uL	73.4%	234079
RDW [He.]	≥15.5%	22.55%	234,022
Hematocrit [He.]	≤40%	67.77%	238,567
INR PT [Co.]	≥1.1 ratio	46.15%	137,973
Bilirubin, Total [Me.]	≥1.5 mg/dL	10.36%	75,016
PT [Co.]	≥12.5 sec	54.11%	137,917
Lymphocytes [Im.]	≤18%	42.77%	171,441
MCHC [He.]	≤31 g/dL	8.61%	234,103

Table S4. Label prevalence (Prev.) and sample count (Counts) for the labels investigated in the main manuscript across the tasks laboratory values abnormality prediction. Next to each value name, we highlight the physiological category it represents: Cardiac (Ca.), Renal (Re.), Hematological (He.), Metabolic (Me.), Immunological (Im.), and Coagulation (Co.). The values in bold represent the highest performance per abnormality. For PT at the 30 minute horizon no score was estimated as it did not satisfy the inclusion criterion of at least 10 abnormal cases in both test and validation set.

Value	Threshold	30m. Prev./Counts	60m. Prev./Counts	120m. Prev./Counts
NTproBNP [Ca.]	≥353 pg/mL	78.93% / 11672	77.47% / 17398	75.95% / 23174
Hemoglobin [He.]	≥17.5 g/dL	0.38% / 105483	0.34% / 149731	0.31% / 197263
Hematocrit [He.]	≥51%	0.62% / 104851	0.56% / 149341	0.52% / 197743
Urea Nitrogen [Re.]	≤6 mg/dL	0.81% / 104076	0.8% / 152037	0.88% / 203206
Creatinine [Re./Ca.]	≥1.2 mg/dL	28.53% / 104595	28.46% / 152946	28.43% / 204543
Urea Nitrogen [Re.]	≥20 mg/dL	41.83% / 104076	41.71% / 152037	41.47% / 203206
Red Blood Cells [He.]	≥6.1 m/uL	0.34% / 103427	0.3% / 146919	0.29% / 193343
Hemoglobin [He.]	≤13.7 g/dL	70.59% / 105483	71.71% / 149731	72.90% / 197263
Albumin [Me.]	≤3.5 g/dL	22.96% / 23560	22.47% / 34015	22.87% / 46419
RDW-SD [He.]	≥46.3 fL	49.56% / 27755	48.85% / 44519	48.54% / 65870
Absolute Monocyte Count [Im.]	≤0.2 K/uL	3.49% / 20660	3.24% / 33509	3.20% / 49559
RDW [He.]	≥15.5%	21.86% / 103402	22.02% / 146880	22.49% / 193284
Absolute Lymphocyte Count [Im.]	≥ 3.7 K/uL	3.46% / 20660	3.06% / 33509	2.87% / 49552
Red Blood Cells [He.]	≤4.6 m/uL	71.71% / 103427	72.48% / 146919	73.37% / 193343
C-Reactive Protein [Im.]	≥ 5 mg/L	63.09% / 1303	58.63% / 2195	57.95% / 3241
Hematocrit [He.]	≤40%	66.08% / 104851	66.98% / 149341	68.03% / 197743
Absolute Basophil Count [Im.]	≤0.01 K/uL	5.63% / 20660	5.27% / 33509	5.11% / 49556
INR PT [Co.]	≥1.1 ratio	44.25% / 60491	45.51% / 83393	46.88% / 109475
Lymphocytes [Im.]	≥42%	5.71% / 81887	5.58% / 114732	5.65% / 147267
PT [Co.]	≥12.5 sec	53.6% / 60409	54.09% / 83310	54.65% / 109393
Lymphocytes [Im.]	≤18%	42.54% / 81887	41.66% / 114732	41.11% / 147267
Anion Gap [Me.]	≥20 mEq/L	8.09% / 100624	7.64% / 146795	7.55% / 196396
MCHC [He.]	≤31 g/dL	11.3% / 103446	10.69% / 146935	10.16% / 193356
Bilirubin Total [Me.]	≥1.5 mg/dL	10.06% / 30934	9.76% / 44888	9.83% / 60625
